# Physical and psychological job demands and fatigue experience among offshore workers

**DOI:** 10.1016/j.heliyon.2023.e16441

**Published:** 2023-05-23

**Authors:** Ahmad Bazazan, Yousuf Noman, Hadis Norouzi, Azam Maleki-Ghahfarokhi, Parvin Sarbakhsh, Iman Dianat

**Affiliations:** a Department of Occupational Health and Ergonomics, Faculty of Health, Tabriz University of Medical Sciences, Tabriz, Iran; b Department of Management, Occupational Health and Safety, University of California, Los Angeles, USA; c Kermanshah Health Centre, Kermanshah University of Medical Sciences, Kermanshah, Iran; d Tabriz University of Medical Sciences, Tabriz, Iran; e Department o Statistics and Epidemiology School of Public Health, Tabriz University of Medical Sciences Tabriz, Iran

**Keywords:** Industrial workers, Mental fatigue, Physical factors, Psychosocial factors, Working conditions

## Abstract

Offshore operations are generally challenging and hazardous, and the workers are exposed to conditions that may lead to fatigue. A cross-sectional study evaluated physical and psychological job demands and their associations with fatigue among offshore workers. The offshore workers (n = 251) completed a questionnaire including demographic/job details, Multidimensional Fatigue Inventory (MFI‒20), and Job Content Questionnaire (JCQ). Data were analysed using linear regression modelling. Results indicated that the physical (particularly performing repetitive motions and applying pressure with hands and wrists) and psychological (e.g., intense task concentration and fast working) job demands were relatively high. The total mean (SD) fatigue score (MFI‒20) was 56.3 (13.9). Individual factors (Body Mass Index ‒ BMI), physical job demands (awkward working postures, frequent moving/lifting heavy objects and doing lots of physical efforts) and psychological job demands (task interruptions by other people and doing an excessive amount of work) were the main variables associated with different dimensions of fatigue in the multivariate models. While physical fatigue was only associated with the physical job demands, both physical and psychological job demands were significantly associated with mental fatigue in the multivariate context. The findings have possible implications for job design and implementation of intervention programmes to promote health and performance of the employees.

## 1 Introduction

In any working environment, there are wide varieties of factors that have potential adverse effects on physical and psychological health of employees [[1], [2], [3], [4]]. Workload is one the main determinants in this regard, which can be classified as physical (e.g., awkward working postures, excessive physical effort and repetitive movements) and psychological (e.g., fast working, time pressure, conflicting demands and task interruptions) demands [2,5]. Fatigue is a serious and common workplace issue that results from a variety of lifestyle and workplace factors [[6], [7], [8]]. According to the National Safety Council, 13% of workplace injuries are related to fatigue and about 97% of workers are exposed to at least one workplace fatigue risk factor [9]. The results of a survey study among manufacturing workers in the United States showed a fatigue prevalence of about 58% among the respondents [10]. Relatively high levels of fatigue have also been reported in other parts of the world [11,12]. As a multidimensional phenomenon (including physical, mental), fatigue can be defined as the subjective experience of weakness, tiredness, and energy deficit [1], influencing health, safety and performance of employees in almost all occupations and countries [1,10,[13], [14], [15], [16]]. Fatigue-related outcomes can be classified as acute (e.g., decreased strength, muscle fatigue) and chronic (e.g., musculoskeletal symptoms, chronic-fatigue syndrome) [13]. A prevalence rate of acute fatigue from 47.8% to 69%, and chronic fatigue from 22.7% to 50.7% has been reported among workers in different occupational groups [17].

A growing body of literature suggests that advanced manufacturing systems impose relatively high levels of physical and mental workloads on workers, leading to a higher prevalence of fatigue [10]. Additionally, due to the multidimensional nature of fatigue, measures or scales that consider multiple aspects of fatigue are preferable. It, therefore, appears to be logical to evaluate the relationships of physical and psychological job demands on different aspects of fatigue. Nevertheless, a review of the literature indicates that relatively limited research has been conducted on this issue in occupational settings. Based on a study among 8833 employees in the United States, it was shown that physical and psychological demands at work increased the risk for fatigue in men [18]. Sembajwe et al. reported significant association between psychosocial components (over-commitment, social support and rewards) and fatigue among 20625 employees in the United States [19]. Hystad et al. found that psychological demands were associated with three aspects of fatigue (physical fatigue, mental fatigue and lack of energy) in seafarers [20]. Parhizi et al. reported strong associations between psychosocial job demands and dimensions of fatigue (mental, physical and total fatigue) [21].

In addition, some studies have also examined the relationship of lifestyle and individual factors with fatigue in the working population [[22], [23], [24]]. Bültmann et al. studied the relationship of lifestyle factors with fatigue among Dutch male employees, and found that being overweight was associated with onset of fatigue in their participants [25]. There are also studies which have found no association between body mass index (BMI) and fatigue among industrial workers [22,23]. Further epidemiological studies might be useful for better understanding of these relationships and planning preventive measures to reduce or prevent fatigue in the workplace.

The oil and gas industry is one of the most important industries in many countries, including Iran. A considerable portion of oil and gas resources is lying under the sea, creating an offshore industry. The offshore industry has been recognised as one of the most hazardous industries globally [26,27]. It has been estimated that the fatality rate among workers in the oil and gas extraction industry is about 7 times higher than the workers' average in the United States [28]. Offshore operations are characterised by prolonged working hours, shift work, limited work spaces (e.g., poor working postures) and living conditions (e.g., insufficient opportunities for rest, shared cabin accommodation), highly repetitive tasks (e.g., maintenance operations), unpleasant environmental conditions (e.g., inappropriate lighting, noise and thermal conditions), chemical hazards (e.g., gases, vapours, fumes, aerosols), fire and explosion hazards, and electrical hazards [27,29,30]. Offshore workers have to cope with such health-threatening conditions during their routine work. As a consequence, workers in the oil and gas extraction industry are subject to relatively high levels of fatigue [28,31]. To the authors’ knowledge, there is little that is known about the occurrence of fatigue and its contributing factors among the offshore workers. The results of a recent review study revealed that offshore workers may be exposed to relatively higher levels of fatigue as compared to workers in other occupations, such as nursing/medical personnel or petrochemical workers [17]. The review recommended focusing on evaluation of fatigue-related risk factors in future studies. Therefore, it was decided in this study to evaluate the relationship of various physical and psychological job demands with different dimensions of fatigue in offshore workers.

Based on the above-mentioned background, the aims of the study were to: 1) determine the physical and psychological job demands in the offshore work environment 2) determine the prevalence of different dimensions of fatigue among offshore workers, 3) evaluate the associations of individual factors and physical and psychological job demands with different aspects of fatigue in offshore workers.

## 2 Methods

### 2.1 Study design and setting

A cross-sectional study was conducted on offshore oil rigs in southwest Iran. After obtaining the ethical approval and required permissions from the concerned authorities, one of the authors (AB) visited the research sites for data collection. The study protocol was approved by the ethical committee of the Health, Safety, and Environment (HSE) Department of Pars Oil and Gas Company (POGC) [Code: IRPOGC. REC.A62/E]. Data were collected via anonymous questionnaires, which took approximately 20 min to complete. The work time duration for the study sites was from 6.00 a.m. to 18.00 p.m. and from 18.00 p.m. to 6.00 a.m., and the shift schedule followed 14 work days and 14 rest days.

### 2.2 Participants

There were approximately 300 workers in the study sites, of which 251 declared their agreement to take part in the study. This represented a response rate of 83%. Having at least 1 year of job experience, no physical disability or mental disease, and not taking any medications, particularly antidepressant or sleeping pills were considered as inclusion criteria for the study [32]. All workers were familiarised with the study aim and procedure before participation. They were told that their participation was voluntary and that they could withdraw from the study at any time. Workers were assured that their responses would be kept confidential. A written informed consent form was signed by each worker before participation.

### 2.3 Data collection

A questionnaire was used for data collection. Demographic details included age (years), weight (kg), height (cm), educational level (primary, secondary, associate, bachelor, masters), marital status (single, married), smoking habits (no, yes) and work experience (years).

Based on the multidimensional nature of fatigue [28], the Multidimensional Fatigue Inventory (MFI‒20) [33] was used for evaluation of fatigue in the study. The MFI–20 is a reliable and validated tool, which has been successfully adapted for measurement of fatigue among industrial workers [[34], [35], [36]]. Validity and reliability of the MFI in Persian is also well established [37]. The main advantage of this tool over other existing tools is that it evaluates different dimensions of fatigue. The MFI–20 evaluates five dimensions of fatigue including general fatigue (4 items), physical fatigue (4 items), reduced motivation (4 items), mental fatigue (4 items), and reduced activity (4 items). Items were rated on a 5-point Likert scale (from (1) = yes, that's correct to (5) = no, that's incorrect). The total possible score for each dimension ranged from 4 to 20, with higher scores indicating a higher level of fatigue. The revised Persian version of the MFI–20 was used [3,34].

Evaluation of perceived physical and psychological job demands was based on the valid, reliable and widely used Job Content Questionnaire (JCQ) [38]. Additional occupation-specific physical demand items were also included, following the recommendations found in the literature [2,39]. The final questionnaire consisted of 18 items including 10 items on physical and 8 items on psychological job demands. Items were rated using a 4-point scale (ranging from 1 = strongly disagree to 4 = strongly agree). This questionnaire has been translated and revised into the Persian language, with established validity and reliability [2]. This revised version was used.

### 2.4 Statistical analysis

Data were analysed using the SPSS v.17 software (SPSS Inc., Chicago, IL, USA). Cronbach's α and Pearson's correlation coefficients were used to assess the internal consistency and correlation among the MFI‒20 and its dimensions. Normality of the data was checked and approved by the Kolmogorov–Smirnov test. The relationships between the occurrence of fatigue (different dimensions of fatigue) and study variables (demographic/job factors and physical and psychological job demands) were initially assessed by univariate linear regression analyses. Those variables with p ≤ 0.05 in the univariate regression analysis were then entered into the multivariate models using stepwise linear regression analysis [40]. For this analysis, five different regressions models were developed for evaluation of different dimensions of fatigue. In the models, the fatigue dimensions were considered as dependent variables and the physical, psychological and individual factors were considered as independent variables. Standardized regression coefficients (β), their corresponding 95% confidence intervals (CIs) and explanatory power (adjusted R square - R2) were calculated for each model. P values less than 0.05 were considered as statistically significant.

## 3 Results

### 3.1 Description of the study population

Table 1 shows the individual data and work-related factors of the study population. The mean age of the participants was 32.5 (range = 22–53; SD = 4.2) years. Their job experience ranged between 2 and 18 years (mean = 8.9 years; SD = 2.8). The majority of participants were married (92.3%) and had a mean (SD) BMI of 26.3 (3.1) kg/m^2^ (range = 17.3–34.9). Approximately one third of the participants (31.7%) had an academic education level.

**Table 1:** Demographic and job characteristics of the study population (*n* = 251).

Variables	
Age (year)	
Mean ± SD	32.5 ± 4.2
Range	22–53
Height (cm)	
Mean ± SD	174.5 ± 6.7
Range	147.0–192.0
Weight (kg)	
Mean ± SD	80.5 ± 1.8
Range	44.0–115.0
BMI (kg/m^2^)	
Mean ± SD	26.3 ± 3.1
Range	17.3–34.9
Marital status (%)	
Single	7.7
Married	92.3
Educational level (%)	
Primary	1.2
Secondary	67.1
Associate	10.5
Bachelor	16.3
Master	4.9
Smoking (%)	
Yes	22.7
No	77.3
Work experience (year)	
Mean ± SD	8.9 ± 2.8
Range	2–18

### 3.2 Physical and psychological demands

Table 2 shows the ratings of the physical and psychological demands. Generally, a large number of participants indicated that physical and psychological demands in their jobs were relatively high (e.g., agree and strongly agree on the scale). In this regard, the most frequent physical demands reported by the respondents were performing repetitive motions with hands/wrists (88.7%) and applying pressure with hands/fingers (88.4%). Intense concentration on the task (89.4%), doing an excessive amount of work (87.5%) and working very fast (82.3%) were the psychological demands most commonly reported by the respondents. The highest and lowest mean scores among the physical job demands were recorded for performing repetitive motions with hands/wrists and pushing/pulling heavy objects with 2.33 and 3.56, respectively. On the other hand, intense concentration on the task (mean score = 3.35) and conflicting demands that others make (mean score = 2.42) were the psychological job demands with the highest and lowest mean scores.

**Table 2:** Ratings of physical and psychological demands.

Variables	Respondents (%)				Mean ratings (SD)
	Strongly agree	Agree	Disagree	Strongly disagree	
**Physical job demands**					
Doing lots of physical efforts (q1)	47.9	27.4	20.1	4.6	3.18 (0.91)
Doing rapid and continuous physical activity (q2)	46.3	31.7	17.1	4.9	3.19 (0.89)
Frequent moving/lifting heavy loads (q3)	14.9	31.7	33.8	19.5	2.42 (0.96)
Working with body in awkward positions (q4)	39.0	40.2	14.3	6.4	3.11 (0.88)
Lifting or lowering objects to/from floor (q5)	26.2	20.7	32.3	20.7	2.52 (1.09)
Lifting or lowering objects to/from shoulder height (q6)	26.5	22.0	26.8	24.7	2.50 (1.12)
Pushing/pulling heavy objects (q7)	16.5	29.3	25.9	28.4	2.33 (0.98)
Standing in one place/static position (>30 min) (q8)	27.7	26.2	25.9	20.1	2.61 (0.96)
Performing repetitive motions with hands/wrists (q9)	70.4	18.3	8.2	3.0	3.56 (0.77)
Applying pressure with hands/fingers (q10)	67.4	21.0	9.1	2.4	3.53 (0.76)
**Psychological job demands**					
Working very hard (q11)	49.7	29.3	18.3	2.7	3.25 (0.85)
Working very fast (q12)	51.8	30.5	15.5	2.1	3.32 (0.81)
Doing an excessive amount of work (q13)	46.3	41.2	11.3	1.2	3.33 (0.72)
Intense concentration on the task (q14)	47.3	42.1	8.8	1.8	3.35 (2.96)
Not having enough time to get the job done (q15)	30.5	39.9	25.3	4.3	2.96 (0.85)
Conflicting demands that others make (q16)	12.2	33.5	39.0	15.2	2.42 (0.89)
Task interruptions by other people (q17)	14.3	44.5	29.0	12.2	2.61 (0.87)
Waiting on work from other people or departments (q18)	34.1	36.9	24.7	4.3	3.01 (0.87)

### 3.3 Fatigue

The total fatigue score of the MFI‒20 for the study population was 56.3 (range = 20–100; SD = 13.9). The general fatigue (mean = 14.8; SD = 3.5) and reduced activity (mean = 8.1; SD = 3.1) were the dimensions with highest and lowest mean scores, respectively. The Pearson's correlation analysis indicated low to moderate positive correlations among the dimensions of the MFI‒20, with r values ranging from 0.134 to 0.645 (Table 3). The correlation coefficients for the total MFI‒20 and its dimensions ranged from 0.514 to 0.852 (Table 3). The Cronbach's α values for the MFI‒20 and its dimensions showed good internal consistency for the MFI‒20 scale, as a whole, and for its dimensions, individually (Table 3).

**Table 3:** Cronbach's α, scores and Pearson correlation coefficients for the Multidimensional Fatigue Inventory (MFI–20) and its dimensions.

MFI scale	Cronbach's α	Mean	SD	Min	Max	(1)	(2)	(3)	(4)	(5)
(1) General fatigue	0.69	14.8	3.5	4	20					
(2) Physical fatigue	0.70	12.3	4.0	4	20	0.615**				
(3) Reduced activity	0.56	8.1	3.1	4	20	0.134*	0.222**			
(4) Reduced motivation	0.57	9.7	3.4	4	20	0.516**	0.582**	0.397**		
(5) Mental fatigue	0.75	11.3	4.0	4	20	0.568**	0.574**	0.641**	0.645**	
(6) Total MFI	0.87	56.3	13.9	20	100	0.762**	0.802**	0.514**	0.823**	0.852**

*p < 0.05 (two-tailed).

**p < 0.01 (two-tailed).

### 3.4 Regression models

The results of multiple linear regression models evaluating the associations between study variables and different dimensions of fatigue (Table 4) are described below. The goodness of fit of the final linear regression models was checked using the residual normality and residual variance constancy tests and no violations of model assumptions were found.

**Table 4:** Factors associated with fatigue dimensions in the multiple regression models.

Fatigue dimensions	Variables retained in the model	B	SE	standard β	95% CI		*P*-value
General fatigue							
	Frequent moving/lifting heavy loads	1.27	0.43	0.17	0.43	2.11	0.003
	Working with body in awkward positions	1.44	0.51	0.16	0.45	2.45	0.005
	Task interruptions by other people	1.26	0.39	0.17	0.50	2.03	0.001
Physical fatigue							
	Frequent moving/lifting heavy loads	1.05	0.48	0.13	0.09	2.00	0.031
	Working with body in awkward positions	2.06	0.59	0.21	0.90	3.22	0.001
	BMI	0.21	0.07	0.17	0.08	0.35	0.002
Reduced activity							
	Doing an excessive amount of work	−1.64	0.60	−0.15	−2.82	−0.45	0.007
	Task interruptions by other people	0.88	0.35	0.14	0.19	1.57	0.013
Reduced motivation							
	Task interruptions by other people	1.54	0.38	0.22	0.79	2.28	<0.001
	Education	−1.20	0.42	−0.16	−2.03	−0.37	0.005
Mental fatigue							
	Doing lots of physical efforts	1.59	0.53	0.17	0.55	2.62	0.003
	Working with body in awkward positions	1.47	0.56	0.15	0.36	2.57	0.009
	Task interruptions by other people	1.63	0.43	0.2	0.77	2.49	<0.001
	BMI	0.2	0.06	0.15	0.06	0.33	0.004
Total fatigue							
	Working with body in awkward positions	7.01	1.88	0.21	3.31	10.73	<0.001
	Task interruptions by other people	6.14	1.47	0.22	3.24	9.04	<0.001
	BMI	0.73	0.23	0.17	0.27	1.19	0.002
	Education	−3.97	1.64	−0.13	−7.2	−0.73	0.017

#### 3.4.1 General fatigue

Both physical and psychological job demands were associated with general fatigue. Frequent moving/lifting heavy objects (β = 0.17, 95% CI: 0.43 to 2.11, *p* < 0.01), working with the body in awkward positions (β = 0.16, 95% CI: 0.45 to 2.45, *p* < 0.01) and task interruptions by other people (β = 0.17, 95% CI: 0.50 to 2.03, *p* < 0.01) were associated with general fatigue. About 12% of the variance in general fatigue was accounted for by variables in the model (R^2^: 0.124).

#### 3.4.2 Physical fatigue

The BMI and two physical job demands including frequent moving/lifting heavy objects (β = 0.13, 95% CI: 0.09 to 2.00, p < 0.05) and working with body in awkward positions (β = 0.21, 95% CI: 0.90 to 3.22, p < 0.001) were associated with physical fatigue. The model explained 12% of the variance in physical fatigue (R2: 0.1).

#### 3.4.3 Reduced activity

Two psychological job demands including doing an excessive amount of work (β = −0.15, 95% CI: −2.82 to −0.45, p < 0.01) and task interruptions by other people (β = 0.14, 95% CI: 0.19 to 1.57, p < 0.05) had significant associations with reduced activity. However, variables in this model did not account for any significant variance in reduced activity (R2 = 0.032).

#### 3.4.4 Reduced motivation

Associations of education and task interruptions by other people (β = 0.22, 95% CI: 0.79 to 2.28, p < 0.001) with reduced motivation were found to be significant. In this model, 14% of the variance in reduced motivation was accounted for the variables assessed (R2: 0.116).

#### 3.4.5 Mental fatigue

The BMI together with physical and psychological factors were associated with mental fatigue. Significant physical and psychological job demands were: working with the body in awkward positions (β = 0.15, 95% CI: 0.36 to 2.57, p < 0.01), doing lots of physical efforts (β = 0.17, 95% CI: 0.55 to 2.62, p < 0.01) and task interruptions by other people (β = 0.2, 95% CI: 0.77 to 2.49, p < 0.001). These variables explained 15% of the variance in mental fatigue (R2: 0.145).

#### 3.4.6 Total fatigue

The BMI, education, working with the body in awkward positions (β = 0.21, 95% CI: 3.31 to 10.73, p < 0.001) and task interruptions by other people (β = 0.22, 95% CI: 3.24 to 9.04, p < 0.001) had significant associations with total fatigue, explaining 19% of the variance in the final model (R2: 0.166).

## 4 Discussion

The present study evaluated the physical and psychological job demands and their associations with the occurrence of fatigue among offshore workers, and the results showed relatively high levels of both physical (particularly performing repetitive motions and applying pressure with hands and wrists) and psychological (e.g., intense task concentration and fast working) job demands in this occupation. Specifically, high physical demands could possibly be attributed to the repetitive work with manual hand tools, particularly wrenches, and tests for monitoring hazardous gases, while working in awkward positions. Similarly, high psychological demands may be attributed to the limited work and life spaces at offshore sites, limitations related to personnel transport to these sites, and potentially dangerous working conditions of offshore workers. These findings highlight the importance of attention to both physical and psychological job demands when developing intervention strategies aimed at improving working conditions of offshore workers. Additionally, it was found that both physical and mental aspects of fatigue were prevalent among the study population. This finding is in part similar to the findings of previous studies conducted in this occupational group [32,41]. This is perhaps not surprising, because it may reflect the reaction of the studied workers to the relatively high levels of physical and psychological job demands in their job. The results also confirmed the relationships of the physical and psychological job demands with different dimensions of fatigue. According to the multivariate models, awkward working postures, frequent moving/lifting heavy objects and doing lots of physical efforts were the main physical job factors, and task interruptions by other people and doing an excessive amount of work were the main psychological job factors associated with fatigue in the studied offshore workers. Two individual factors including BMI and education were also associated with the occurrence of fatigue in the multivariate context. These findings highlight the role of individual factors together with physical and psychological job demands in the experience of fatigue in this occupation. The findings may have implications in terms of job design and implementation of intervention programmes to promote health and performance of the employees.

The findings clearly indicate the need for comprehensive intervention programmes to improve working conditions and reduce fatigue-related exposures. It is obvious that employees can do their tasks better and more safely when they experience less fatigue. The mean total fatigue score (e.g., mean score of the MFI‒20) in our study was 56.3, which is much higher than those reported for the Colombian (43) [42], or German general populations (41.1) [43]. This value is also much higher than those reported for some industrial workers such as petrochemical employees (42) [34] and for hospital nurses (43 to 50) [3,44]. Higher fatigue scores among the offshore workers in comparison with the general populations or other occupational groups reveals the challenging nature of this job. With regard to the adverse effects of fatigue on employee's health, performance and safety outcomes [14,45], it is necessary to take actions to reduce this problem. In this regard, workplace (e.g., planning work-rest schedules, shift work scheduling, and job re-design) and lifestyle (e.g., adequate quantity and quality of sleep, doing exercise and physical activity, social interactions) interventions have been shown to be effective fatigue management and mitigation strategies in the literature [10,45].

As shown in this research, both physical and psychological job demands were associated with the experience of fatigue among the studied offshore workers. This finding is perhaps not surprising in view of previous studies, which have shown positive associations of physical [25] and psychological [[19], [20], [21],^25^] job demands with different dimensions of fatigue among workers in different occupations. However, several points should be noted here. First, the different dimensions of fatigue were not affected the same way by the physical and psychological job demands. It is of interest to note that the physical aspect of fatigue was only associated with the physical job demands, while both physical and psychological job demands had significant associations with mental fatigue in multivariate models. This finding suggests that different prevention strategies may be required in order to improve the experience physical and mental fatigue in this working population. Based on this finding, physical job demands should be targeted for improvement of physical fatigue, whereas intervention programmes for prevention of mental fatigue should consider both physical and psychological job demands. Moreover, the reduced activity and reduced motivation aspects of fatigue were only related to the psychological job demands. These findings have practical implications in terms of designing intervention strategies for prevention of fatigue in these employees. Another point that should be noted is that the strength of associations between physical and psychological job demands with different dimensions of fatigue did not vary considerably. In other words, none of the physical or psychological job demands had stronger relationships to different aspects of fatigue (e.g., β values ranged between ±0.13 and ± 0.22). This means that both physical and psychological job demands are equally important when dealing with the issue of fatigue in this population. More specifically, working in awkward positions and task interruptions by other people were the most frequent physical and psychological job demands, respectively, that were associated with different dimensions of fatigue. As discussed earlier, the prominent role of awkward working postures in experiencing fatigue can be attributed to the nature of offshore operations. With regard to the task interruptions, it should be noted that these events are very common in many occupations, and they can be categorised as daily stressors or strains at work [46]. This is because these events not only interrupt the main workflow (e.g., delay in task implementation), but they can also lead to further tasks that have to be performed. Past research has shown positive associations of task interruptions with reduced performance and occurrence of physical pain and discomfort in employees [46,47]. It is, therefore, reasonable to observe that the task interruption was a main independent factor influencing fatigue in offshore workers. The final point that should be made is that, despite consideration of a wide range of physical and psychological job demands, the explanatory power of the models was relatively low (e.g., except the model examining reduced activity, adjusted R-squared values ranged between 0.10 and 0.17). It is therefore plausible to assume that other important factors may have been involved in the experience of fatigue in this working population. Thus, it is necessary in future studies to consider a wider range of variables such as environmental conditions (e.g., noise, lighting and thermal conditions) organisational factors (e.g., organisational support), and individual factors (e.g., sleep patterns, sport/physical activities, nutrition, individual coping measures) that may have a potential to influence fatigue in this group of industrial employees.

Another point that should be noted is that the fatigue management issue is a joint responsibility between workplace management and employees [45]. This means that some fatigue-related preventive measures should be discussed in terms of individual employee involvement. Therefore, individual and lifestyle factors may play a major role in this regard. Several notable findings were found with regard to individual risk factors. BMI and education were independent factors of fatigue in the multivariate models, probably implying the role of lifestyle interventions in the management of fatigue in the workplace. There are mixed findings in the literature with regard to the relationship between BMI and fatigue among industrial workers, with some studies showing a positive association [25] and others showing no association [22,23]. Based on our findings, it can be recommended that, in addition to the physical and psychological job demands, individual factors should be taken into account as potential confounders in future analysis of fatigue complaints in working populations. Employee education and training can also be considered as an effective measure to mitigate fatigue or fatigue-related consequences in employees [7,8].

Regarding the sample size and power of the study, it should be noted that this study was conducted using the census method among the eligible workers (e.g., all eligible workers were asked to participate), and therefore the sample size was not determined. However, the power of the study considering the main objective of the study (e.g., associations of physical and psychological job demands with different aspects of fatigue), the available sample size (n = 251), the minimum observed correlation between and variables (0.2), and Type I error equal to 0.05 and using the Fisher's z-test for comparing correlation with zero was obtained as 0.88, which is a acceptable.

Despite a number of significant contributions, the results should be interpreted in the light of some limitations such as the study design (e.g., cross-sectional as opposed to a longitudinal or experimental methodology) and generalisability of the findings. Therefore, it is recommended that future studies use more objective measures, and include other occupational groups and settings to improve understanding and generalisability of these findings. Consideration of a wider range of variables (including environmental, organisational and individual factors), which may have the potential to contribute to fatigue experience in offshore workers is also recommended.

## 5 Conclusion

This paper presents an overview of the demanding and potentially dangerous nature of the offshore work environment. Workers were generally exposed to relatively high levels of physical and psychological demands in their job, which reveals the challenging nature of this occupation. High levels of different aspects of fatigue were also common among the studied workers, and fatigue levels were shown to be associated with exposure to both strenuous physical and psychological job demands. The findings emphasise the need for workplace and lifestyle interventions addressing individual, physical and psychological factors with the aim of preventing or at least reducing fatigue among workers in this industry.

## Author contribution statement

Ahmad Bazazan, Yousuf Noman, Hadis Norouzi, Azam Maleki-Ghahfarokhi, Parvin Sarbakhsh and Iman Dianat: Conceived and designed the experiments; Performed the experiments; Analysed and interpreted the data; Contributed reagents, materials, analysis tools or data; Wrote the paper.

## Data availability statement

Data will be made available on request.

## Declaration of competing interest

The authors declare that they have no known competing financial interests or personal relationships that could have appeared to influence the work reported in this paper.
