# Supplementary material for: Physical and psychological job demands and fatigue experience among offshore workers
Source: Heliyon. 2023 May 23;9(6):e16441. doi: 10.1016/j.heliyon.2023.e16441 (PMC10238685; doi:10.1016/j.heliyon.2023.e16441)
Supplement: Multimedia component 1 [file mmc1.docx]

**MFI-20**

**Fatigue**

By using this questionnaire, we want to assess your situation in the recent days. In order to complete the questionnaire, please notice to the below example:

**“I have the feeling of calm nerves”**

If you think that this sentence is completely true in your case, it means that you had the feeling of calm nerves in recent days, please put a Celtic cross in the first square, it means square number 1, like the answer below:

Yes, it is completely true 1 **2** **3** **4** **5** no, it is completely wrong

Your more disagreement with the considered sentence, according to the disagreement degree, put the Celtic cross in the direction of “no, it is completely wrong”, please put the Celtic cross for the correct answer for all the sentences and put the Celtic cross just for one answer in every sentence.

| 1 | I feel fit | Yes, it’s completely right | ❑1 | ❑2 | ❑3 | ❑4 | ❑5 | No, it’s completely wrong |
| --- | --- | --- | --- | --- | --- | --- | --- | --- |
| 2 | Physically I feel only able to do a little | Yes, it’s completely right | ❑1 | ❑2 | ❑3 | ❑4 | ❑5 | No, it’s completely wrong |
| 3 | I feel very active | Yes, it’s completely right | ❑1 | ❑2 | ❑3 | ❑4 | ❑5 | No, it’s completely wrong |
| 4 | I feel like doing all sorts of nice things | Yes, it’s completely right | ❑1 | ❑2 | ❑3 | ❑4 | ❑5 | No, it’s completely wrong |
| 5 | I feel tired | Yes, it’s completely right | ❑1 | ❑2 | ❑3 | ❑4 | ❑5 | No, it’s completely wrong |
| 6 | I think I do a lot in a day | Yes, it’s completely right | ❑1 | ❑2 | ❑3 | ❑4 | ❑5 | No, it’s completely wrong |
| 7 | When I am doing something, I  can keep my thoughts on it | Yes, it’s completely right | ❑1 | ❑2 | ❑3 | ❑4 | ❑5 | No, it’s completely wrong |
| 8 | Physically I can take on a lot | Yes, it’s completely right | ❑1 | ❑2 | ❑3 | ❑4 | ❑5 | No, it’s completely wrong |
| 9 | I dread having to do things | Yes, it’s completely right | ❑1 | ❑2 | ❑3 | ❑4 | ❑5 | No, it’s completely wrong |
| 10 | I think I do very little in a day | Yes, it’s completely right | ❑1 | ❑2 | ❑3 | ❑4 | ❑5 | No, it’s completely wrong |
| 11 | I can concentrate well | Yes, it’s completely right | ❑1 | ❑2 | ❑3 | ❑4 | ❑5 | No, it’s completely wrong |
| 12 | I am rested | Yes, it’s completely right | ❑1 | ❑2 | ❑3 | ❑4 | ❑5 | No, it’s completely wrong |
| 13 | It takes a lot of effort to  concentrate on things | Yes, it’s completely right | ❑1 | ❑2 | ❑3 | ❑4 | ❑5 | No, it’s completely wrong |
| 14 | Physically I feel I am in a bad condition | Yes, it’s completely right | ❑1 | ❑2 | ❑3 | ❑4 | ❑5 | No, it’s completely wrong |
| 15 | I have a lot of plans | Yes, it’s completely right | ❑1 | ❑2 | ❑3 | ❑4 | ❑5 | No, it’s completely wrong |
| 16 | I tire easily | Yes, it’s completely right | ❑1 | ❑2 | ❑3 | ❑4 | ❑5 | No, it’s completely wrong |
| 17 | I get little done | Yes, it’s completely right | ❑1 | ❑2 | ❑3 | ❑4 | ❑5 | No, it’s completely wrong |
| 18 | I don’t feel like doing anything | Yes, it’s completely right | ❑1 | ❑2 | ❑3 | ❑4 | ❑5 | No, it’s completely wrong |
| 19 | My thoughts easily wander | Yes, it’s completely right | ❑1 | ❑2 | ❑3 | ❑4 | ❑5 | No, it’s completely wrong |
| 20 | Physically I feel I am in an  excellent condition | Yes, it’s completely right | ❑1 | ❑2 | ❑3 | ❑4 | ❑5 | No, it’s completely wrong |

Please answer (✓ or🗶) the following questions in table

**JCQ**

**Physical and Psychological demands**

| **Physical demand items:** | | | | | | | |
| --- | --- | --- | --- | --- | --- | --- | --- |
| **My job requires:** | | | | | | | |
| 1 | Doing lots of physical efforts | strongly disagree | ❑1 | ❑2 | ❑3 | ❑4 | strongly agree |
| 2 | Doing rapid and continuous physical activity | strongly disagree | ❑1 | ❑2 | ❑3 | ❑4 | strongly agree |
| **In my job, I am:** | | | | | | | |
| 3 | Frequent moving/lifting heavy loads | strongly disagree | ❑1 | ❑2 | ❑3 | ❑4 | strongly agree |
| 4 | Working with body in awkward positions | strongly disagree | ❑1 | ❑2 | ❑3 | ❑4 | strongly agree |
| **How often in a typical workday do you:** | | | | | | | |
| 5 | Lifting or lowering objects to/from floor | strongly disagree | ❑1 | ❑2 | ❑3 | ❑4 | strongly agree |
| 6 | Lifting or lowering objects to/from shoulder height | strongly disagree | ❑1 | ❑2 | ❑3 | ❑4 | strongly agree |
| 7 | Pushing/pulling heavy objects | strongly disagree | ❑1 | ❑2 | ❑3 | ❑4 | strongly agree |
| 8 | Standing in one place/static position (>30 min) | strongly disagree | ❑1 | ❑2 | ❑3 | ❑4 | strongly agree |
| 9 | Performing repetitive motions with hands/wrists | strongly disagree | ❑1 | ❑2 | ❑3 | ❑4 | strongly agree |
| 10 | Applying pressure with hands/fingers | strongly disagree | ❑1 | ❑2 | ❑3 | ❑4 | strongly agree |
| **Psychological demand items:** | | | | | | | |
| **My job requires:** | | | | | | | |
| 11 | Working very hard | strongly disagree | ❑1 | ❑2 | ❑3 | ❑4 | strongly agree |
| 12 | Working very fast | strongly disagree | ❑1 | ❑2 | ❑3 | ❑4 | strongly agree |
| 13 | Doing an excessive amount of work | strongly disagree | ❑1 | ❑2 | ❑3 | ❑4 | strongly agree |
| 14 | Intense concentration on the task | strongly disagree | ❑1 | ❑2 | ❑3 | ❑4 | strongly agree |
| 15 | Not having enough time to get the job done | strongly disagree | ❑1 | ❑2 | ❑3 | ❑4 | strongly agree |
| **My job:** | | | | | | | |
| 16 | Conflicting demands that others make | strongly disagree | ❑1 | ❑2 | ❑3 | ❑4 | strongly agree |
| 17 | Task interruptions by other people | strongly disagree | ❑1 | ❑2 | ❑3 | ❑4 | strongly agree |
| 18 | Waiting on work from other people or departments | strongly disagree | ❑1 | ❑2 | ❑3 | ❑4 | strongly agree |

Please answer (✓ or🗶) the following questions in table.
